# Synergistic Effect of Strontium and Melt Quenching on the Solidification Microstructure of Hypereutectic Al-Si Alloys

**DOI:** 10.3390/ma16186188

**Published:** 2023-09-13

**Authors:** Yunfei Wang, Wenyao Chen, Ya Liu, Haoping Peng, Jianhua Wang, Xuping Su

**Affiliations:** 1 Jiangsu Key Laboratory of Materials Surface Science and Technology, Changzhou University, Changzhou 213164, China; s21010805028@smail.cczu.edu.cn (Y.W.); wenyaochen2010@126.com (W.C.); yliu@cczu.edu.cn (Y.L.); penghp@cczu.edu.cn (H.P.); sxping@cczu.edu.cn (X.S.); 2 Jiangsu Collaborative Innovation Center of Photovoltaic Science and Engineering, Changzhou University, Changzhou 213164, China

**Keywords:** hypereutectic Al-Si alloy, strontium, melt quenching, synergistic effect, solidified microstructure

## Abstract

The synergistic effect between strontium (Sr) and melt quenching on the solidified microstructure of hypereutectic Al-Si alloys was investigated by optical and scanning electron microscopy. The results indicate that melt quenching can suppress the growth of primary Si particles in the solidified structure of the hypereutectic Al-Si alloy, resulting in a significant decrease of in the average size of primary Si particles in Al-(18~22)Si alloys from 30.35~66.31 μm to 15.13~34.63 μm. The synergistic effect between Sr and melt quenching can further inhibit the precipitation of primary Si particles in the Al-18Si alloy. After the addition of Sr to Al-18Si alloy and undergoing melt quenching, the area fraction of primary Si clearly decreases. When the added amount of Sr increases from 0.1 wt.% to 0.5 wt.%, the area fraction of primary Si decreases from 1.13% to 0.16%. With 0.5 wt.% Sr in the tested alloy, the inhibiting effect on primary Si precipitation was significantly improved. Research has shown that the cooling rate has a significant impact on the solidified structure of the melt-quenched Al-18Si-0.5Sr alloy. There exists no primary Si in solidified structures on the area of 1/8R and 1/4R from the surface of the round bar sample, but the area fraction of primary Si increases, respectively, to 1.97% and 12.48% on the area of 1/2R and R from the surface. The higher the cooling rate, the higher the inhibitory effect on the primary Si precipitation in the Al-18Si-0.5Sr alloy.

## 1. Introduction

Hypereutectic Al-Si alloy has advantages such as high specific strength, good thermal conductivity, good airtightness, low thermal expansion coefficient, and good casting performance. It is widely used in fields such as automobiles, aerospace, construction engineering, and electronic communication [1,2,3]. The hypereutectic Al-Si alloys with silicon in the range of 16–24 wt.% are ideal materials for manufacturing cylinders and pistons [4]. As the silicon content increases, the size of primary Si particles in hypereutectic Al-Si alloys gradually increases, exhibiting rough plate-like and angular shapes. Under external forces, microcracks are prone to occur at the sharp corners of the primary Si phase, resulting in a decrease in the strength and plasticity of the alloy [5,6,7], severely limiting the use of hypereutectic Al-Si alloys in industries.

The refinement of primary Si in eutectic Al-Si alloys and the continuous improvement of the comprehensive mechanical properties have always been the focus of material science research. The refining methods of primary Si mainly include electromagnetic stirring [8], ultrasonic treatment [9], modification treatment [10], alloying with trace elements [11], and rapid solidification [12]. Owing to its simplicity and significant effectiveness, modification treatment is most widely used to refine the primary Si in Al-Si alloys [13,14], such as modification with P [15] and RE [16], and complex modification with several elements [17]. Mao Feng et al. [18] studied the effect of P and Eu composite modification onAl-16Si alloy and found that the Eu addition can further refine the primary Si. Meanwhile, lamellar eutectic silicon could be transformed into fibrous eutectic silicon, and the wear resistance of the alloy could be significantly improved. Microalloying can also effectively decrease the size of primary Si in Al-Si alloys. Adding trace elements, for example, Na, Sr, and Sb, to the alloy can change the shape and size of the primary Si particles and can inhibit the growth of the phase. Tebib et al. [19] studied the effect of Sr addition on the primary Si of the Al-15Si-14Mg-4Cu alloy and found that the Sr addition could change the morphology of Si particles from coarse needle to slender fiber. Li et al. [17] studied the effect of Sr, RE, and P ternary compound modification on the solidified structure of Al-20Si and Al-Si alloys. The results showed that the compound modification displayed a certain refinement effect on primary Si and eutectic silicon. In addition, the deep undercooling rapid solidification process can increase the undercooling rate of the alloy melt and inhibit the precipitation of primary Si. Shehata et al. [20] found that the size of primary Si particles decreased from 88.5 to 21.7 μm through the rheocasting of an A390 alloy melt at 690 °C using a water-cooled slope plate. The results showed that the primary Si in the hypereutectic Al-Si alloy can be refined to a certain extent but cannot be eliminated.

Until now, existing research focused mainly on refining primary Si in hypereutectic Al-Si alloys. However, it is difficult to suppress the precipitation of primary Si, and it is even more difficult to eliminate the primary Si in the solidified structure. The novelties of the present research are that the growth of primary Si is suppressed by melt quenching, and the precipitation of primary Si is remarkably inhibited by the synergistic effect of Sr and melt quenching. In addition, a pseudo eutectic structure is obtained near the surface of the alloy sample due to a rapid cooling rate. The research results of this paper can provide valuable references for further improving the microstructure of hypereutectic Al-Si alloys, improving the mechanical properties of pistons and cylinder bodies, and expanding the application of hypereutectic Al-Si alloys in industry.

## 2. Experimental Preparation and Methods

**Figure 1:**
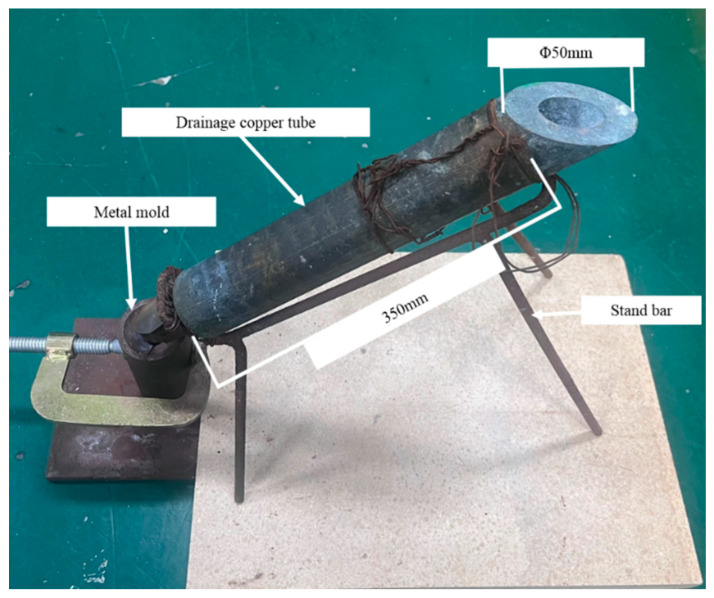
Melt quenching casting device.

High-purity Al (99.5 wt.%), Al-50Si and Al-10Sr master alloys were used in the experiment. Table 1 shows the chemical compositions of the two master alloys used. The particle size of the intermediate alloys is in the range of 5 to 10 mm. To eliminate the adverse effect of primary Si in the Al-50Si alloy, according to a certain proportion, Al-Si (16–24 wt.%) alloys were melted in an SG2-5-12 high-precision temperature-controlled well furnace at 850 °C for 30 min after placing high-purity Al ingot and Al-50Si intermediate alloy in a graphite crucible, respectively. Then, small ingots were prepared by pouring the melt into room temperature metal molds. Specific experimental methods can be found in reference [21]. The melt quenching casting device comprises a bracket and a copper tube as shown in Figure 1. The length of the tube is 350 mm, the inner diameter is 25 mm and the outer diameter is 50 mm. The copper tube was cooled through immersion in liquid nitrogen and then removed to increase its temperature to −160 °C for later use.

This experiment investigates the effect of melt quenching on the solidification structure of hypereutectic aluminum silicon alloy. First, 50 g of Al-Si (16–24 wt.%) alloy was collected and remelted at 850 °C in a well resistance furnace using a graphite crucible and kept for 30 min. The alloy melt was poured into a low-temperature copper tube for rapid cooling and solidified in a mild steel mold (25 °C) with inner sizes of *Φ*12 × 120, and the alloy test bars were obtained for future use. In addition, Al-Si (18, 20, 22 wt.%) alloy melt without rapid cooling was poured into the mild steel mold with inner dimensions of *Φ*12 × 120. The alloy bars obtained were used as reference samples for future use.

To study the synergistic effect between strontium and melt quenching on the solidified structure of hypereutectic Al-Si alloys, 50 g Al-Si (16–24 wt.%) alloy was remelted in a well type resistance furnace at 850 ° C using a graphite crucible, and 0.5 wt.% of Al-10Sr master alloy was added and then held for 30 min. To study the effect of strontium content on the melt-quenched alloys, 0.1 wt.% and 0.3 wt.% of Al-10Sr master alloys were added to the Al-18Si alloy. These alloy melts were poured into a low-temperature copper tube and the mild steel mold with inner sizes of *Φ*12 × 120, and then the test rods were obtained for later experimental use.

The alloy at the center of the round bar sample exhibits the slowest cooling rate. The farther away from the center of the bar sample, the faster the cooling rate of the alloy. By observing and analyzing the microstructure of various parts at different distances from the center of the sample, the cooling rate effect on the solidified structure of the melt-quenched Al-18Si-0.5Sr alloy was studied.

The metallographic specimens were prepared according to reference [21]. Keller reagent (95 mL H_2_O, 2.5 mL HNO_3_, 1.5 mL HCl, 1.0 mL HF) was used for corrosion. The solidified structures of 1/8R, 1/4R, 1/2R and R from the edge of the alloy round bar samples were observed using a Leica DMI 3000 metallographic microscope. The instrument operating parameters used in this experiment were the same as in the literature [21], and the primary Si and α-Al phases of the alloy were quantitatively analyzed using an Image-Pro Plus 6.0 software. The alloy phases were analyzed using a Smartlab X-ray diffractometer, and the microstructure and composition of the alloy specimen were examined using a JSM-6360LV scanning electron microscope. The microstructure and phase compositions of the alloy were further analyzed using a JSM-6360LV scanning electron microscope (JEOL, Tokyo, Japan) equipped with an energy-dispersive X-ray spectrometer (Oxford Instruments, Oxford, UK). The spectral working distance was 15 mm and the acceleration voltage was 20 kV.

## 3. Experimental Results and Discussion

### 3.1. Effect of Melt Quenching on the Solidified Structure of Hypereutectic Al-Si Alloys

**Figure 2:**
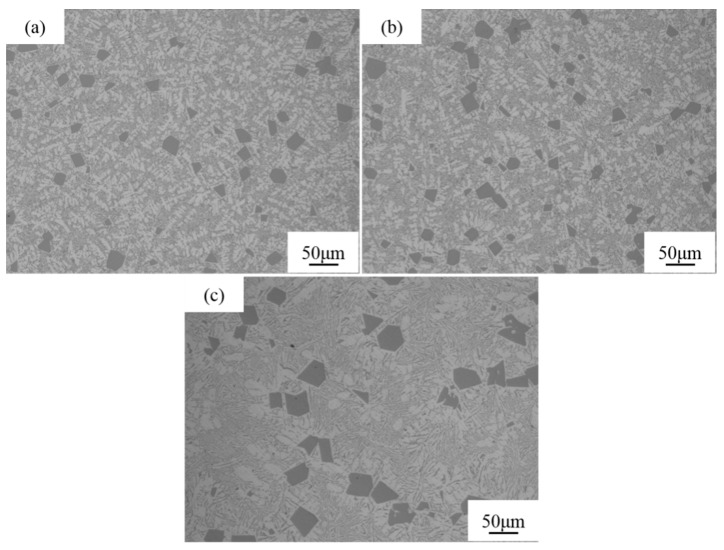
Solidification structure of Al-*x*Si alloy. (**a**) *x* = 18, (**b**) *x* = 20, (**c**) *x* = 22.

Figure 2 shows the solidified structures of Al-Si (18–22 wt.%) alloys without melt quenching. The hypereutectic Al-Si alloy comprises black irregular bulky primary Si particles, white dendritic α-Al phase, and lamellar (α-Al + Si) structure. At a silicon content of 18 wt.%, the Al-Si alloy features small numbers and sizes of primary Si particles and exhibits a fine dendritic α-Al phase as shown in Figure 2a. As the amount of silicon is increased by 20 wt.%, the number and size of primary Si particles in the Al-Si alloy slightly increase, and the dendritic α-Al phase becomes slightly coarse as shown in Figure 2b. When the silicon content is 22 wt.%, the number of the primary Si particles in the alloy significantly decreases, the particle size significantly increases, and the α-Al phase is coarse granular as shown in Figure 2c. Based on quantitative analysis, the size of primary Si in the solidification structure of Al-18Si, Al-20Si and Al-22Si alloys is 30.35, 32.61 and 66.31 μm, respectively. According to the Al-Si phase diagram, as the silicon content increases, the temperature range of the solid-liquid two-phase zone increases. There is enough time for primary Si to grow, so the size of primary Si particles in the alloy increases.

**Figure 3:**
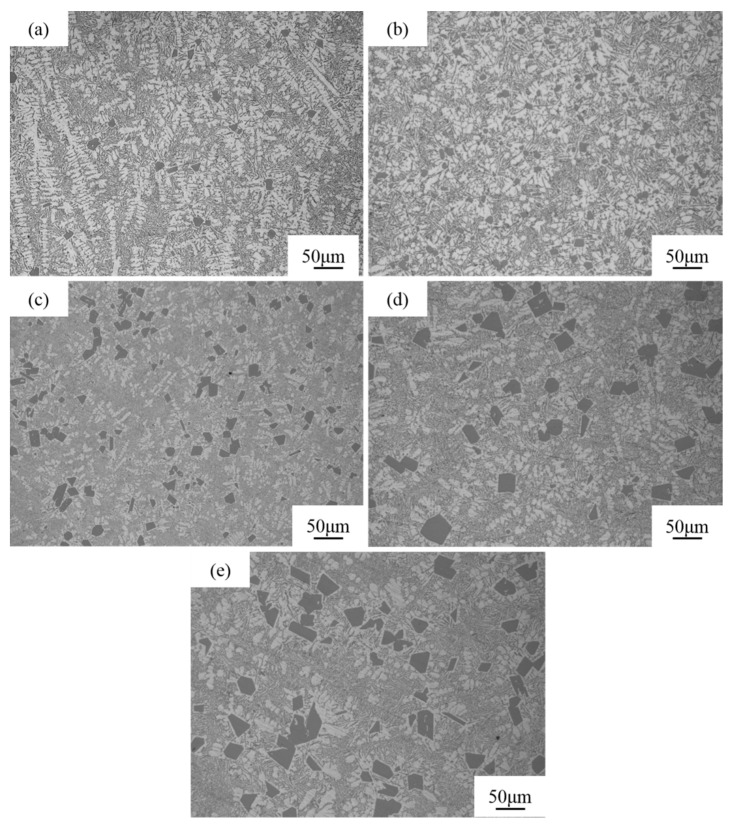
Solidification structure of melt-quenched Al-*x*Si alloy. (**a**) 16 wt.% Si, (**b**) 18 wt.% Si, (**c**) 20 wt.% Si, (**d**) 22 wt.% Si, (**e**) 24 wt.% Si.

Figure 3 shows the solidified structures of melt-quenched Al-Si (16–24 wt.%) alloys. At a high silicon content of 16 wt.%, the alloys display a small amount of fine primary Si particles and a large number of dendritic α-Al phases as shown in Figure 3a. As the silicon amount is increased to 18 wt.%, the sizes of primary Si particles in the alloys are slightly changed, but the number of primary Si particles significantly increases. In addition, the number of α-Al phases in the alloys slightly decreases, and the dendritic α-Al phases are weakened as shown in Figure 3b. The solidification microstructure of the Al-18Si alloy without melt quenching exhibits larger numbers of primary Si particles and lower particle sizes as shown in Figure 2a. As the silicon content increases to 20 wt.%, the number of primary Si particles in the alloy remarkably decreases, but the size is remarkably increased. In addition, the alloy features lower numbers of refined α-Al phases and dendritic α-Al phase characteristics as shown in Figure 3c. The solidification microstructure of the Al-20Si alloy without melt quenching exhibits higher numbers of primary Si and lower particle sizes as shown in Figure 2b. As the silicon content increases to 22 wt.%, the size of coarse Si particles in the alloy significantly increases, and the number of particles remarkably decreases. In addition, the number of dendritic α-Al phase in the alloy decreases and the phase becomes slightly coarse as shown in Figure 3d. The solidification microstructure of the Al-22Si alloy without melt quenching features significantly large numbers of primary Si particles and small particle sizes as shown in Figure 2c. As the amount of silicon increases to 24 wt.%, the number and the size of primary Si in the alloy slightly increase, and the dendritic α-Al phase is slightly coarsened as shown in Figure 3e. In summary, the melt-quenched hypereutectic Al-Si alloys display significantly large numbers of primary Si particles and small particle sizes, indicating that melt quenching can effectively promote the nucleation of primary Si during the solidification of the hypereutectic Al-Si alloy and inhibit its growth.

Table 2 displays the quantitative analysis results of the size of primary Si particles in the solidified structure of the melt-quenched hypereutectic Al-Si alloy. The primary Si particles in the alloy feature an average size of 14.63 μm at a silicon content of 16 wt.% as shown in Table 2. With increasing silicon content, the average size of primary Si particles in the alloy gradually increases. As the silicon content increases from 18 to 20 wt.%, the average size of primary Si particles in the alloy significantly increases. With increasing silicon content, the area fraction of primary Si particles in the alloy gradually increases, and the area fraction of the α-Al phase decreases. The comprehensive analysis results reveal that melt quenching features the greatest inhibitory effect on the growth of primary Si particles in the Al-18Si alloy.

**Figure 4:**
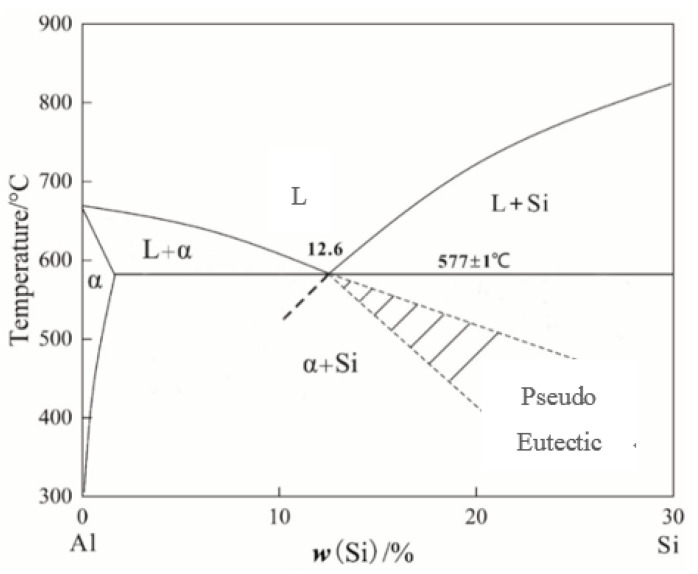
Schematic phase diagram of Al-Si alloy.

The influence mechanism of melt quenching on the solidified structure of the Al-18Si alloy is elucidated as follows. Figure 4 shows a schematic phase diagram of the Al-Si alloy. The liquidus temperature of the alloy gradually increases with increasing silicon content. Thus, because the hypereutectic Al-Si alloy is melted at 850 °C, the superheat of the alloy melt gradually decreases as the silicon content increases from 16 to 24 wt.%. Due to their structural property, more or less primary Si residual particles should be present in the Al-Si alloy melt. With the increase of Si content, the superheat of the alloy melt decreases, and the number and size of residual Si particles in the alloy melt gradually increase. Therefore, despite the use of melt-quenching processes to accelerate the cooling rate of the melt, primary Si particles are still present in the solidification microstructure. With increasing silicon content in the Al-Si alloy, the number and size of primary Si particles in the solidified alloy structure increase. In addition, with increasing silicon content in the Al-Si alloy, the primary Si particles in the alloy melt increase, which inhibits the nucleation and growth of the α-Al phase and results in the decrease of the α-Al phase area fraction.

### 3.2. Effect of Strontium and Melt Quenching on Solidified Structure of the Al-18Si Alloy

**Figure 5:**
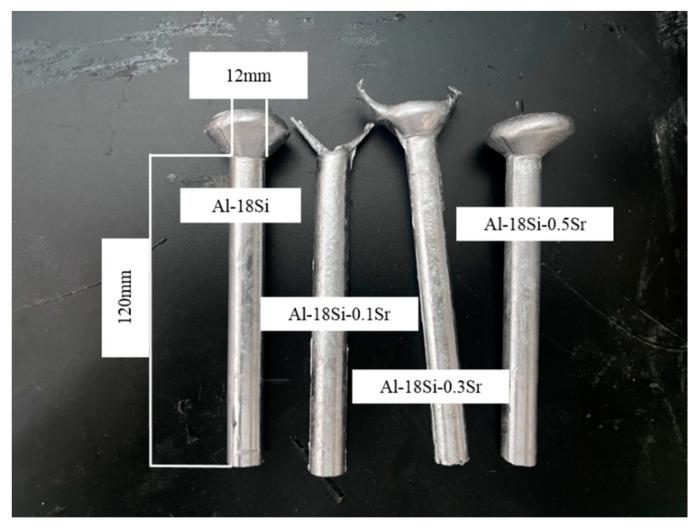
Melt-quenched Al-18Si-*x*Sr alloy samples.

**Figure 6:**
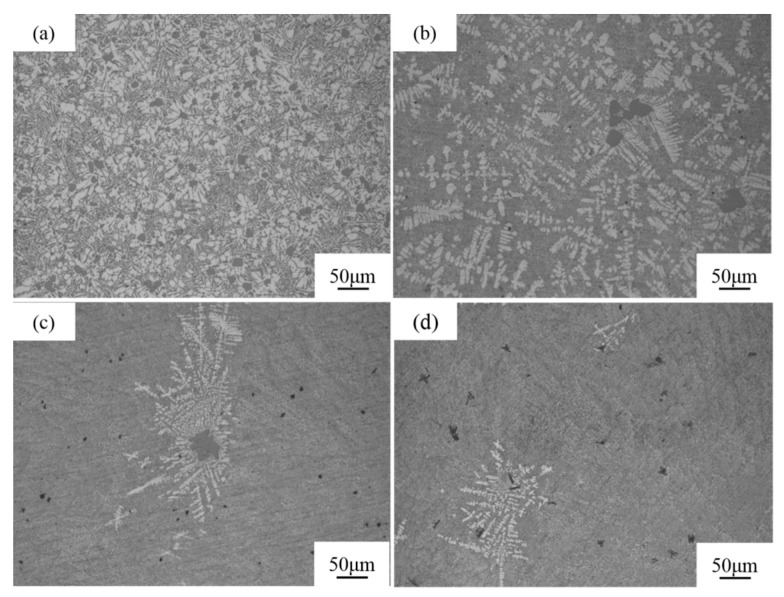
Solidification structure of melt-quenched Al-18Si-*x*Sr alloy. (**a**) 0.0 wt.% Sr, (**b**) 0.1 wt.% Sr, (**c**) 0.3 wt.% Sr, (**d**) 0.5 wt.% Sr.

The melt-quenched Al-18Si-xSr alloy samples are shown in Figure 5. The sizes of the samples are *Φ*12 × 120. Figure 6 shows the solidified microstructure of the melt-quenched Al-18Si-*x*Sr alloy. The solidified structure of the melt-quenched Al-18Si alloy indicates that owing to the addition of 0.1 wt.% Sr to the alloy, the melt-quenched alloy exhibits significantly small numbers of primary Si particles and large particle sizes, as shown in Figure 6a. In addition, the melt-quenched alloy displays small numbers of primary dendritic α-Al phase and features a refined lamellar eutectic structure. With 0.3 wt.% strontium, the alloy features small numbers of coarse primary Si particles and α-Al phases in dendritic forms, which are mainly gathered around the primary Si as shown in Figure 6b. In addition, the alloy displays a small amount of black strontium-containing compound particles as shown in Figure 6c. When the Sr content is 0.5 wt.%, the coarse primary Si particles in the alloy disappear, but there are only a small amount of primary Si particles with small sizes, and the amount of α-Al phase further decreases and distributes round the primary Si. In addition, the alloy features significantly large sizes of black particles as shown in Figure 6d. The analysis results reveal that the synergistic effect between of Sr and melt quenching can significantly inhibit the precipitation of primary Si in the Al-18Si alloy and decrease the area fraction of primary Si particles. With increasing strontium content, its inhibition effect on the precipitation of primary Si is continuously enhanced. However, when the amount of Sr is more than 0.3 wt.%, black strontium-containing compound particles are formed in the Al-18Si alloy.

**Figure 7:**
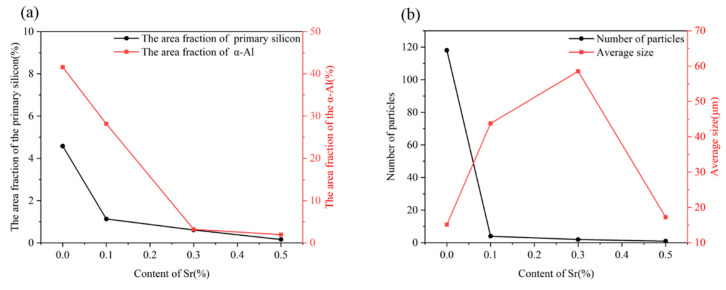
Relationship curve between quantitative results of phase and Sr content in melt-quenched Al-18Si-*x*Sr alloy. (**a**) Area fraction of primary Si/α-Al phase as a function of Sr content. (**b**) The number/average size of primary Si as a function of Sr content.

Table 3 displays the quantitative analysis results of the solidified structure of the melt-quenched Al-18Si alloy. Figure 7 shows the quantitative analysis curves of the constituent phases in the melt-quenched Al-18Si-*x*Sr alloy as a function of Sr content. Without the addition of Sr, the primary Si particles and the α-Al phase in the melt-quenched Al-18Si alloy exhibit an area fraction of 4.58%, and 41.57%, respectively, as shown in Table 3. Due to the addition of Sr to the Al-18Si alloy, the area fraction of primary Si particles and α-Al phase significantly decrease. With increasing Sr content, the area fraction of primary Si particles and α-Al phase decrease as shown in Figure 7a. The number of primary Si particles in the alloy significantly decreases due to the addition of strontium to the Al-18Si alloy as shown in Figure 7b. With increasing strontium content, the number of primary Si particles in the alloy continuously decreases. In addition, after the addition of strontium to the Al-18Si alloy, the primary Si particles feature large sizes. With increasing strontium content, the size of primary Si particles in the alloy first increases and then decreases. These results show that strontium can inhibit the nucleation of primary Si, and its inhibition effect increases with increasing strontium content. In addition, the average size of primary Si particles in the hypereutectic Al-Si alloy increases after the strontium addition, indicating that strontium cannot inhibit the growth of primary silicon particles in the melt-quenched Al-18Si alloy.

**Figure 8:**
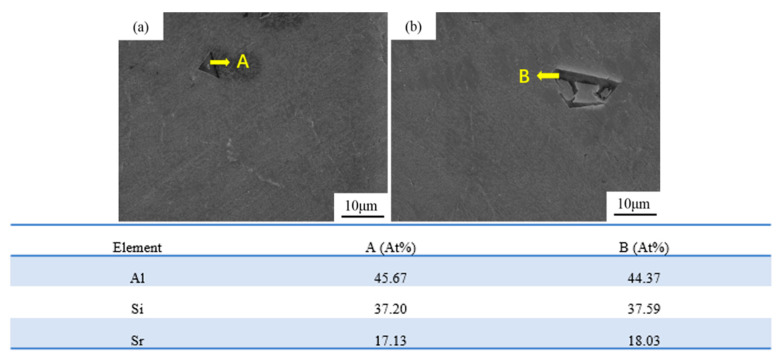
Energy spectrum analysis of Sr compounds in melt-quenched Al-18Si-*x*Sr. (**a**) 0.3 wt.%, (**b**) 0.5 wt.%.

Figure 8 shows the results of energy spectrum analysis for the particles in melt-quenched Al-18Si-xSr alloy. The analysis results (Figure 8a,b) reveal that the melt-quenched alloy displays irregular angular black compound particles. The combined results of this energy spectrum analysis and reports from the literature [21] show that the black compound particles are present in the Al_2_Si_2_Sr phase. With increasing strontium content, the structure of the black compounds remains unchanged, and the particle size in the Al_2_Si_2_Sr phase increases. The formation of the Al_2_Si_2_Sr phase significantly decreases the precipitation temperature of primary Si. The high undercooling process effectively inhibits the nucleation of primary Si and reduces the number of primary Si particles in the solidification microstructure of the alloy. Owing to the small number of primary Si particles, the high silicon content in the surrounding alloy melt could provide abundant Si atoms to promote the coarsening of primary Si particles, thereby significantly increasing their sizes [21].

### 3.3. Effect of Cooling Rate on Solidified Structure of the Melt-Quenched Al-18Si-0.5Sr Alloy

**Figure 9:**
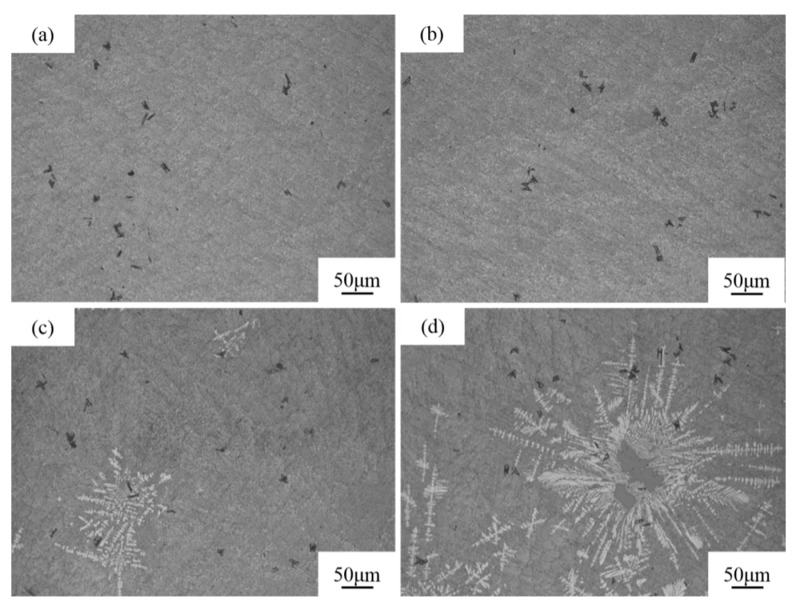
Solidification microstructure of various parts at different distances from the center of the sample of Al-18Si-0.5Sr alloy. (**a**) 1/8R, (**b**) 1/4R, (**c**) 1/2R, (**d**) R.

Figure 9 shows the solidified structure of various parts at different distances from the center of the sample of the melt-quenched Al-18Si-0.5Sr alloy. No primary Si particles and α-Al phases are present in the solidified structure of the melt-quenched Al-18Si-0.5Sr alloy at 1/8R and 1/4R position from the edge surface of the round bar sample, but only the eutectic structure and Al_2_Si_2_Sr phase exist as shown in Figure 9a,b. The primary Si particles disappear because of the higher cooling rate. At 1/2R from the edge of the bar sample, the solidified structure of the Al-18Si-0.5Sr alloy features fine black primary Si particles and a small amount of dendritic α-Al phase as shown in Figure 9c. The solidified structure at the center of the melt-quenched Al-18Si-0.5Sr alloy is shown in Figure 9d. Compared with the solidified structure of the melt-quenched alloy at 1/2R position, the size of primary Si particles in the solidified structure at the center of the alloy is significantly increased, and the particles exist in the form of irregular blocks and clear edges. The size and the area fraction of primary Si increase because of the lower cooling rate. There is large number of the α-Al phase around the primary Si. Each part of the sample features a different cooling rate, and the closer the alloy to the edge of the sample, the higher the cooling rate of the melt. The analysis results indicate that with decreasing cooling rate, the size of primary Si particles in the solidified structure of the melt-quenched Al-18Si-0.5Sr alloy is larger, and the area fraction of the α-Al phase is larger, too.

**Figure 10:**
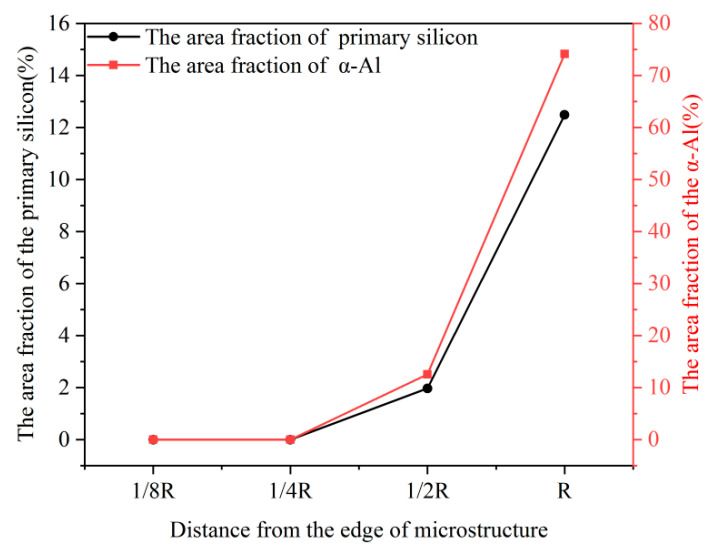
The area fraction of primary Si and α-Al phase in melt-quenched Al-18Si-0.5Sr alloy as a function of position.

Table 4 displays the quantitative analysis results of the solidified structure of different parts of the melt-quenched Al-18Si-0.5Sr alloy. Figure 10 shows the area fraction of primary Si and α-Al phase in melt-quenched Al-18Si-0.5Sr alloy as a function of position. No primary Si particle is present in the positions at 1/8R and 1/4R from the edge of the sample as shown in Table 4 and Figure 9. From 1/2R to the center of the bar sample, the area fraction of primary Si particles in the alloy increases from 1.97% to 12.48%, and the average size of primary Si particles increases from 12.75 to 73.77 μm. The quantitative analysis results show that the cooling rate has a great influence on the solidified structure of the melt-quenched Al-18Si-0.5Sr alloy. The higher the cooling rate, the higher the inhibiting effect on the precipitation and growth of primary Si particles.

## 4. Conclusions

(1) Melt quenching can restrain the growth of primary Si in the solidified structure of the hypereutectic Al-Si alloy and significantly decrease the average size of primary Si particles. Melt quenching exhibited the highest inhibiting effect on the growth of primary Si particles in the Al-18Si alloy. The average size of primary Si particles in Al-(18~22)Si alloys decreases from 30.35~66.31 μm to 15.13~34.63 μm.
(2) The complementary effect between Sr and melt quenching can effectively inhibit primary Si from precipitation in the Al-18Si alloy. When the Sr content is 0.1, the number of primary Si particles begins to decrease. At a high Sr content of 0.3 wt.%, the Al-18Si alloy exhibits an Al_2_Si_2_Sr phase, resulting in a further decrease in primary Si particles. In addition, the size of primary Si particles shows a trend of first increasing and then decreasing. At a strontium content of 0.5 wt.%, its inhibition effect on the precipitation of primary Si was significantly enhanced, and the size of primary Si was the smallest. The area fraction of primary Si in melt-quenched Al-18Si alloy is 4.58%, which clearly decreases from 1.13 to 0.16% when the added amount of Sr in the alloy increases from 0.1 to 0.5 wt.%.
(3) The cooling rate has a great influence on the solidified structure of the melt-quenched Al-18Si-0.5Sr alloy. At areas of 1/8R and 1/4R from the edge surface of the bar sample, no primary Si appears because of the higher cooling rate. From 1/2R to the center R, the area fraction of primary Si increases because of the lower cooling rate. A higher cooling rate can significantly restrain the precipitation and growth of primary Si particles in hypereutectic Al-Si Alloys.

## Figures and Tables

**Table 1:** Chemical compositions of the used master alloys.

Master Alloy	Chemical Compositions (wt.%)						
	Si	Fe	Cu	Sr	Mn	Mg	Al
Al-50Si master alloy	50	0.3	0.1	-	0.1	0.1	Bal.
Al-10Sr master alloy	0.1	0.3	0.1	10	0.1	0.1	Bal.

**Table 2:** Quantitative analysis results of primary Si size in solidification microstructures of melt-quenched hypereutectic Al-Si alloys.

Si/wt.%	Average Size/(μm)	Maximum Size/(μm)	Minimum Size/(μm)
16	14.63	22.75	6.50
18	15.13	19.50	10.75
20	29.63	49.50	9.75
22	34.63	62.00	7.25
24	35.25	64.01	6.40

**Table 3:** Quantitative analysis results of melt-quenched Al-18Si-*x*Sr alloy.

Sr/wt.%	The Area Fraction of Primary Si/%	The Area Fraction of α-Al Phase/%	Average Size of Primary Si/μm
0	4.58	41.57	15.13
0.1	1.13	28.16	43.75
0.3	0.61	3.19	58.51
0.5	0.16	1.97	17.25

**Table 4:** Quantitative analysis results of melt-quenched Al-18Si-0.5Sr alloy at different positions.

Different Locations	The Area Fraction of Primary Si/%	Average Size of Primary Si/μm
1/8R	—	—
1/4R	—	—
1/2R	1.97	12.75
R	12.48	73.77

## Data Availability

The raw/processed data required to reproduce these findings cannot be shared at this time due to legal or ethical reasons.
